# Targeting *KRAS* Oncogene in Colon Cancer Cells with 7-Carboxylate Indolo[3,2-*b*]quinoline Tri-Alkylamine Derivatives

**DOI:** 10.1371/journal.pone.0126891

**Published:** 2015-05-29

**Authors:** Hugo Brito, Ana Cláudia Martins, João Lavrado, Eduarda Mendes, Ana Paula Francisco, Sofia A. Santos, Stephan A. Ohnmacht, Nam-Soon Kim, Cecília M. P. Rodrigues, Rui Moreira, Stephen Neidle, Pedro M. Borralho, Alexandra Paulo

**Affiliations:** 1 Cell Function and Therapeutic Targeting Group, Research Institute for Medicines (iMed.ULisboa), Faculty of Pharmacy, Universidade de Lisboa, Av. Prof. Gama Pinto, 1649-003 Lisbon, Portugal; 2 Medicinal Chemistry Group, Research Institute for Medicines (iMed.ULisboa), Faculty of Pharmacy, Universidade de Lisboa, Av. Prof. Gama Pinto, 1649-003 Lisbon, Portugal; 3 Faculty of Pharmacy, Universidade de Lisboa, Av. Prof. Gama Pinto, 1649-003 Lisbon, Portugal; 4 Center for Systems Biology, Massachusetts General Hospital, Boston, MA 02114, United States of America; 5 UCL School of Pharmacy, University College London, 29/39 Brunswick Square, London WC1N 1AX, United Kingdom; 6 Medical Genomics Research Center, Korea Research Institute of Bioscience and Biotechnology, Daejeon, 305-333, Republic of Korea; Winship Cancer Institute of Emory University, UNITED STATES

## Abstract

**Background:**

A guanine-rich strand within the promoter of the *KRAS* gene can fold into an intra-molecular G-quadruplex structure (G4), which has an important role in the regulation of *KRAS* transcription. We have previously identified indolo[3,2-*b*]quinolines with a 7-carboxylate group and three alkylamine side chains (IQ3A) as effective G4 stabilizers and promising selective anticancer leads. Herein we investigated the anticancer mechanism of action of these compounds, which we hypothesized due to stabilization of the G4 sequence in the *KRAS* promoter and subsequent down-regulation of gene expression.

**Methodology/Principal Findings:**

IQ3A compounds showed greater stabilization of G4 compared to duplex DNA structures and reduced *KRAS* promoter activity in a dual luciferase reporter assay. Moreover, IQ3A compounds showed high anti-proliferative activity in HCT116 and SW620 colon cancer cells (IC_50_ < 2.69 μM), without eliciting cell death in non-malignant HEK293T human embryonic kidney, and human colon fibroblasts CCD18co. IQ3A compounds significantly reduced *KRAS* mRNA and protein steady-state levels at IC_50_ concentrations, and increased p53 protein steady-state levels and cell death by apoptosis in HCT116 cells (mut *KRAS*, wt *p53*). Furthermore, KRAS silencing in HCT116 p53 wild-type (p53(+/+)) and null (p53(-/-)) isogenic cell lines induced a higher level of cell death, and a higher IQ3A-induced cell death in HCT116 p53(+/+) compared to HCT116 p53(-/-).

**Conclusions:**

Herein we provide evidence that G4 ligands such as IQ3A compounds can target G4 motifs present in *KRAS* promoter, down-regulate the expression of the mutant *KRAS* gene through inhibition of transcription and translation, and induce cell death by apoptosis in colon cancer cell lines. Thus, targeting KRAS at the genomic level with G4 ligands may be a new anticancer therapy strategy for colon cancer.

## Introduction

The *KRAS* gene encodes a G-protein which serves as a molecular switch between the endothelial growth factor receptor and the nucleus, controlling several signaling pathways important for cell growth and survival. KRAS GTPase exists in two states, a GTP-bound active state and a GDP-bound inactive state. Further, *KRAS* mutations increase KRAS affinity for GTP leading to the constitutive activation of the protein. Importantly, deletion of mutant *KRAS* allele in colon cancer cell lines dramatically reduces cellular proliferation [1], highlighting the fact that many tumors harboring mutant *KRAS* are KRAS-dependent. *KRAS* mutations are mostly prevalent in pancreatic (90–60%), colorectal (30–50%) and lung (20–30%) carcinomas [2]. Due to the high incidence of these cancers worldwide [3] and the increased resistance to conventional chemotherapy [4], the search for new targets has been intensified in past recent years.

The importance of therapeutic modulation of KRAS signaling have been widely recognized and several approaches have been reported in the past, but none has provided an approved new anticancer drug to date [5]. An innovative therapeutic approach being studied is the use of miRNAs, since they play an important role in chemo-sensitization [6]. We have previously demonstrated that miRNA-143, which also reduces *KRAS* expression, chemosensitizes colon cancer cells to 5-fluorouracil [7], and reduces tumor growth *in vivo*, with increased apoptosis and reduced proliferation [8].

Continuing our studies that aim to discover novel and selective anticancer drugs, pursued through the development of several chemical systems to be used in diverse therapeutic strategies [9], [10],[11], we report here on an approach to targeting KRAS signaling in cancer cells by directly modulating *KRAS* expression at the gene level. It has been recently demonstrated that a guanine-rich strand within the promoter of *KRAS* can fold into an intra-molecular G-quadruplex structure (G4), which has an important role in the regulation of *KRAS* transcription [12],[13]. G4 arrangements are nucleic acid higher-order structures, formed by sequences containing repetitive guanine(G)-rich tracts [14]. Several studies have provided evidence supporting the existence of G4s in eukaryotic telomeres and oncogene promoters, including those of *KRAS*, *HRAS*, *HSP90*, *c-MYC*, *c-KIT*, *BCL-2* and *VEGF* genes, and that small molecules stabilizing G4 structures are able to down-regulate oncogene transcription in tumor cell lines, inhibit telomerase activity and induce cancer cell growth arrest [15],[16],[17]. G4 structures have also been found in RNA sequences, including in the 5′ untranslated region (UTR) of *KRAS* mRNA, and shown to have translation regulatory functions [18],[19],[20].

**Fig 1:**
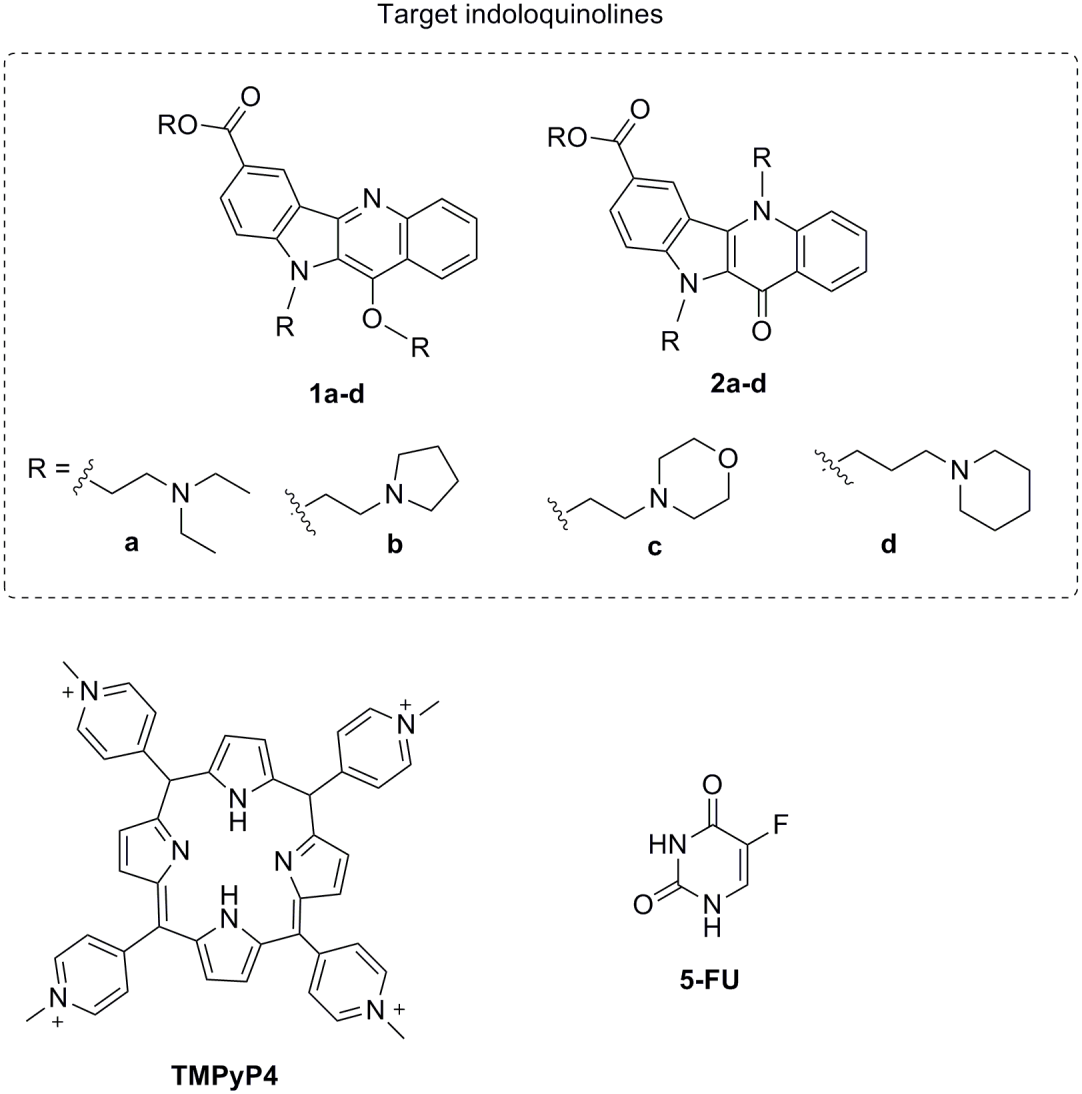
Structures of studied compounds. 7-carboxylate indolo[3,2-*b*]quinoline tri-alkylamine derivatives (IQ3A) **1a-d** and **2a-d**; G4 ligand porphyrin derivative TMPyP4 and 5-fluorouracil (5-FU) anticancer drug.

Indoloquinolines are natural alkaloids able to target DNA structures, some of which have potential for development into anticancer drugs [21],[22]. Indolo[3,2-*b*]quinoline derivatives have been shown to be potent G4 ligands, and to inhibit cell proliferation and oncogene (*c-MYC*) transcription [21],[23],[24]. Moreover, we have recently discovered that indolo[3,2-*b*]quinolines with a 7-carboxylate group and three alkylamine side chains (**1a** and **2a** in Fig 1) are promising selective anticancer leads [25]. These compounds selectively inhibited (100-fold) the growth of *KRAS* mutant HCT116 colon cancer cells compared to primary rat hepatocytes, while also decreasing KRAS protein levels. In order to exploit this scaffold towards the discovery of novel and improved anticancer drugs, we have extended the chemical diversity of these indoloquinolines and studied their potential anticancer mechanism of action. Previous structure-activity studies with mono-alkylamine indolo[3,2-*b*]quinolines have shown that optimal G4 stabilization was induced by compounds with propyl side chains and basic amine groups (p*Ka* ≥ 8) [25]. Thus, compounds **1a-d** and **2a-d** (Fig 1) were designed, synthesized and evaluated for selective G4 thermal stabilization comparing to duplex DNA, together with inhibition of cancer cell proliferation, induction of apoptosis and down-regulation of *KRAS* and *HSP90* transcription and protein expression. In order to improve the anticancer activity profile and *KRAS* oncogene down-regulation capacity of our target indoloquinolines, we have used four cell lines with differing *KRAS* and *TP53* genotypes, as well as two positive controls, the anticancer drug 5-fluorouracil (5-FU) and the G4 ligand TMPyP4 (Fig 1).

## Results and Discussion

Compounds **1a-d** and **2a-d** were synthesized in four steps following the procedure previously described [25] with some modifications (S1 Text). Structures of **1a-d** and **2a-d** were completely elucidated by bidimensional ^1^H (COSY and NOESY) and ^13^C heterocorrelation NMR experiments (HMQC and HMBC) and purity (> 95%) confirmed by HPLC-ELSD-MS (S1 Fig).

**Fig 2:**
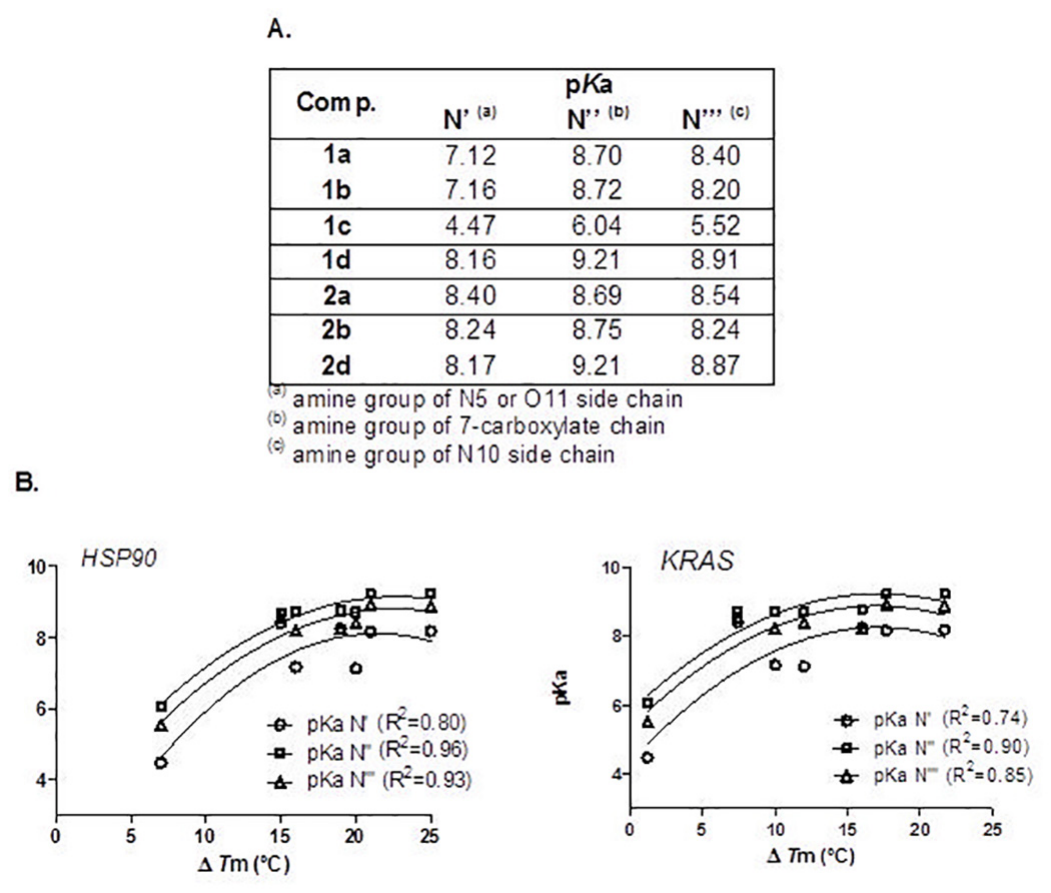
Basicity of side chains correlates positively with thermal G4 stabilization. **A.** Calculated p*K*a values of side chain amine groups by SPARC (v. 4.6). **B.** Plots of variation of G4 thermal stabilization by compounds (Δ*T*_m_) with basicity (p*K*a) of side chains groups.

The capacity of compounds **1a-d**, **2a-d** and the standard G4 ligand TMPyP4 (Fig 1) [16] to bind and stabilize *KRAS* [26] and *HSP90A* [27] G4 DNA structures as well as duplex DNA (T-loop) was evaluated by a Fluorescence Resonance Energy Transfer (FRET) melting assay. The increase in the melting temperatures induced by different concentrations of compounds is presented in Table 1 and S2 Fig. Our results show that tri-alkylamine indolo[3,2-*b*]quinolines (IQ3A) are potent and selective ligands for the *KRAS* and *HSP90A* G4 structures. As previously observed for mono- and di-alkylamine analogues [25] and other polyaromatic-fused G4 ligands [28],[29], compounds with propylamine side chains (**1d** and **2d**) are superior G4 stabilizers (Δ*T*_m_ between 18 and 23°C at 2 μM of ligand) than compounds with shorter alkylamine side chains (**1a-b** and **2a-b**; Δ*T*_m_ values between 7 and 17°C at 2 μM ligand concentration). It was observed that the basicity of side chains correlates positively with thermal G4 stabilization of all DNA sequences up to an optimal pKa ~ 8.0–9.0 (Fig 2). Heterocyclic amines at the end of alkyl side chain (**1b** and **2b**) appear to improve binding to G4s and complex stabilization compared to the di-ethylamine group (**1a** and **2a**), which cannot be explained by differences in basicity between terminal groups. Additionally, and in line with what was previously observed for di-alkylamine indoloquinolines [25], this study suggests that N5,N10,COO tri-substitution with heterocyclic amine groups at alkyl side chain termini increase ligand affinity to G4, as **2b,d**-G4 DNA complexes have higher melting temperatures than complexes of the correspondent isomers **1b,d**. However, this was not observed for the isomeric pair **1a** / **2a**. Despite the increased G4 stabilization capacity, ligands **2b** and **2d** were not able to significantly discriminate between the two DNA G4 structures (Table 1).

**Table 1:** Melting temperatures (Δ*T*m) of DNA G-quadruplex (KRAS21, HSP90) and duplex (T-Loop) structures stabilized by compounds 1a-d, 2a-d and TMPyP4 at 1 and 2 μM.

Comp.	Δ*T*m (°C)^(a)^					
	KRAS21		HSP90A		T-Loop	
	1 μM	2 μM	1 μM	2 μM	1 μM	2 μM
**1a**	8	12	14	20	<2	3
**1b**	5	10	12	16	<2	6
**1c**	<2	<2	4	7	<2	<2
**1d**	9	18	15	21	<2	10
**2a**	4	7	11	15	<2	<2
**2b**	10	16	14	19	3	6
**2d**	13	22	19	25	4	12
**TMPyP4**	25	n.d.	n.d.	n.d.	16	n.d.

^(a)^ standard deviation < 0.2°C;

n.d. = not determined.

To study the effect of IQ3A compounds on cancer and non-cancer cells, we selected compound **2d** showing the best G4 stabilization capacity *in vitro* and the pair **1a** and **2a**, which despite showing lower Δ*T*_m_ values for the respective complexes with G4 DNA, are able to reduce *KRAS* expression when incubated in HCT116 colorectal cancer cells, as we have previously shown [25].

The short-term effect of compounds at 72 h on cell growth was studied using colorectal carcinoma cell lines with different *KRAS* and *TP53* genotypes: human colorectal carcinoma HCT116 (mut *Kras*, wild-type (wt) *p53*), and human metastatic colorectal adenocarcinoma SW620 (mut *Kras*, mut *p53*). In parallel, we also used the immortalized human embryonic kidney cell line HEK293T (wt *Kras*, wt *p53*) and normal human colon fibroblast cells CCD18co (wt *Kras*, wt *p53*). The IC_50_ and IC_65_ values in Table 2 show that compounds **2a** and **2d** display superior anti-proliferative activity compared to **1a** and **5-FU**, particularly in metastatic SW620 cells, where **2d** gave an IC_50_ value (0.28 μM), almost 20-fold lower than that of the standard anticancer drug 5-FU (IC_50_ = 5.39 μM). Interestingly, colorectal cancer cells HCT116 and SW620, which express mutant KRAS, were particularly insensitive to the porphyrin derivative TMPyP4, in contrast to non-malignant HEK293T cells expressing wild type KRAS. In addition, IQ3A compounds and 5-FU, were not selective for cancer cells expressing mutant KRAS, since they were equally active against HCT116, SW620 and HEK293T cells. Nevertheless, we observed some selectivity (S.I. > 2.4; Table 2) toward the colon cancer cell line HCT116 compared to normal colon fibroblasts (CCD18co).

**Table 2:** Antiproliferative activity (IC_50_ and IC_65_ in μM)^a^ of compounds 1a, 2a, 2d, TMPyP4 and 5-fluorouracil (5-FU) in malignant (HCT116, SW620) and non-malignant (HEK293T, CCD18co) cell lines.

Comp.	HCT116		SW620		HEK293T		CCD18co	SI ^(b)^
	IC_50_	IC_65_	IC_50_	IC_65_	IC_50_	IC_65_	IC_50_	
**1a**	1.88^c^	3.42	2.69	4.12	3.23	6.06	4.63	2.46
**2a**	0.99^c^	1.60	0.67	1.44	0.67	0.94	5.41	5.46
**2d**	0.40	0.58	0.28	0.45	0.70	1.12	2.18	5.45
**TMPyP4**	12.39	14.21	> 20	> 20	2.97	4.11	38.17	3.08
**5-FU**	2.38^c^	4.13	5.39	8.32	2.66	4.13	>100	> 42

^(a)^ Standard errors ≤ 0.1;

^(b)^ Selective index (SI) = IC_50_ CCD-18co / IC_50_ HCT116;

^(c)^ Previously reported IC_50_ of 4.5(**1a**),5.7(**2a**) and 3.79(**5-FU**) μM using different cell culture conditions.[25]

**Fig 3:**
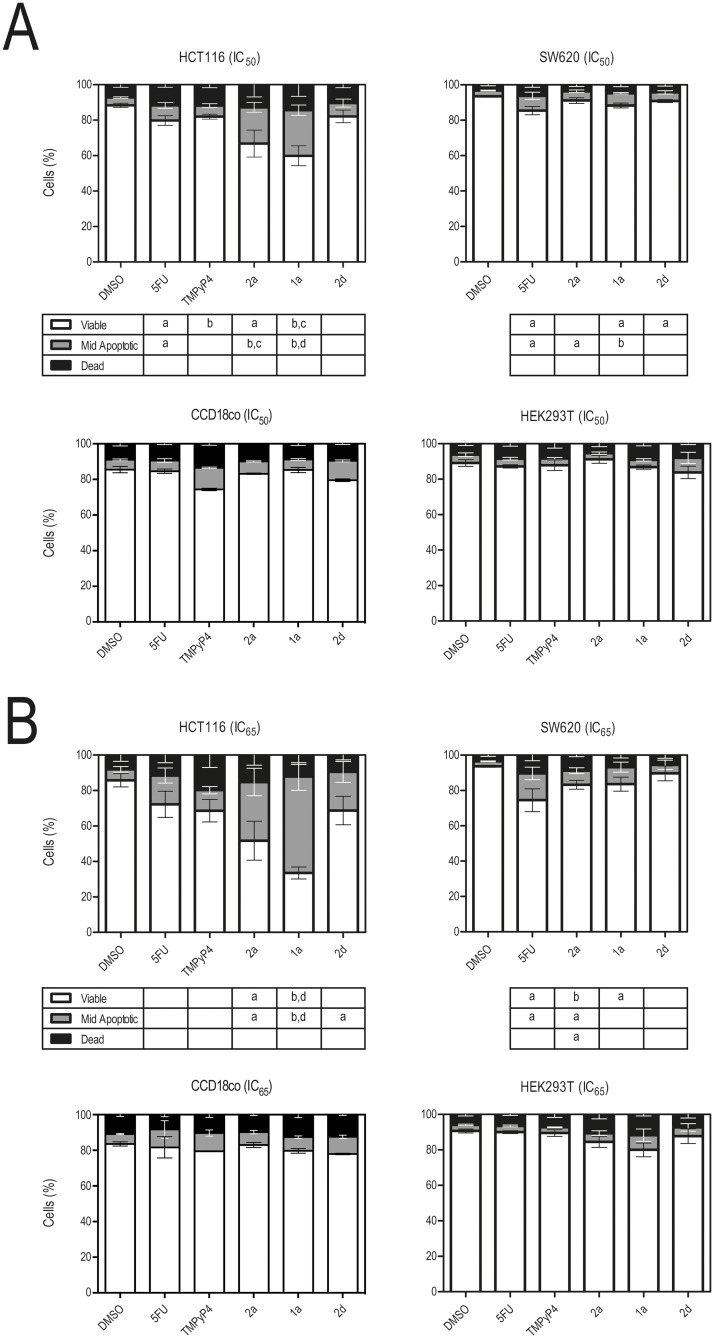
Exposure to IQ3A compounds induces cell death, mainly by apoptosis in HCT116 cells. Cell populations were obtained by Guava ViaCount flow cytometry following 72 h incubation of HCT116 colon cancer, SW620 metastatic colon cancer, CCD18co human colon fibroblasts and HEK293T embryonic kidney cell lines with 5-FU, TMPyP4 and IQ3A at equitoxic (IC_50_ and IC_65_) concentrations, or DMSO (vehicle control). Results are expressed as the mean percentage (%) of viable, mid-apoptotic and dead cells ± SEM, of at least three different experiments, for **A.** IC_50_, and for **B.** IC_65_ compound concentrations. a, p < 0.05; b, p < 0.01 from DMSO (vehicle control); c, p < 0.05; and d, p < 0.01 from 5-FU.

Subsequently, the Guava ViaCount assay was used to evaluate the effects of IQ3A compounds on cell death induction in cancer (HCT116 and SW620) and non-malignant (HEK293T and CCD18co) cells, compared to 5-FU and TMPyP4 at equitoxic concentrations (IC_50_ and IC_65_). Down-regulation of mutant *KRAS* expression by antisense oligonucleotides in colorectal cancer cells [7],[8],[30] and by a MAZ-binding oligonucleotide decoy in pancreatic cancer cells [31] has been associated with increased apoptosis and cell growth arrest. Also, anticancer drugs, such as 5-FU and G4 stabilizers of telomeric and oncogene promoter sequences such as TMPyP4, are known to induce a generalized cell response leading to cell growth arrest and cell death by apoptosis (DNA damage response) [15],[32], which involves activation of the pro-apoptotic transcription factor p53 among other pathways in the case of 5-FU [34]. Fig 3 shows that our test compounds have distinct effects on cells, depending on compound structure and probably also on the genetic background of an individual cell line. In HCT116 cells, all compounds induced cell death by apoptosis, but the IQ3A compounds showed the most significant apoptotic effect, with a trend of **1a** > **2a** > **2d**, and causing 30–70% cell death at concentrations ranging from 0.58 to 3.42 μM (IC_65_) (Fig 3B, upper left panel). Conversely, all compounds were unable to induce significant cell death in HEK293T and CCD18co cells (Fig 3A and 3B, lower panels), whereas in SW620 cells only 5-FU was able to induce apoptosis in a dose-dependent manner (Fig 3A and 3B, upper right panels). The effect of TMPyP4 on SW620 was not studied, as this cell line was not sensitive to this compound up to a 20 μM concentration.

**Fig 4:**
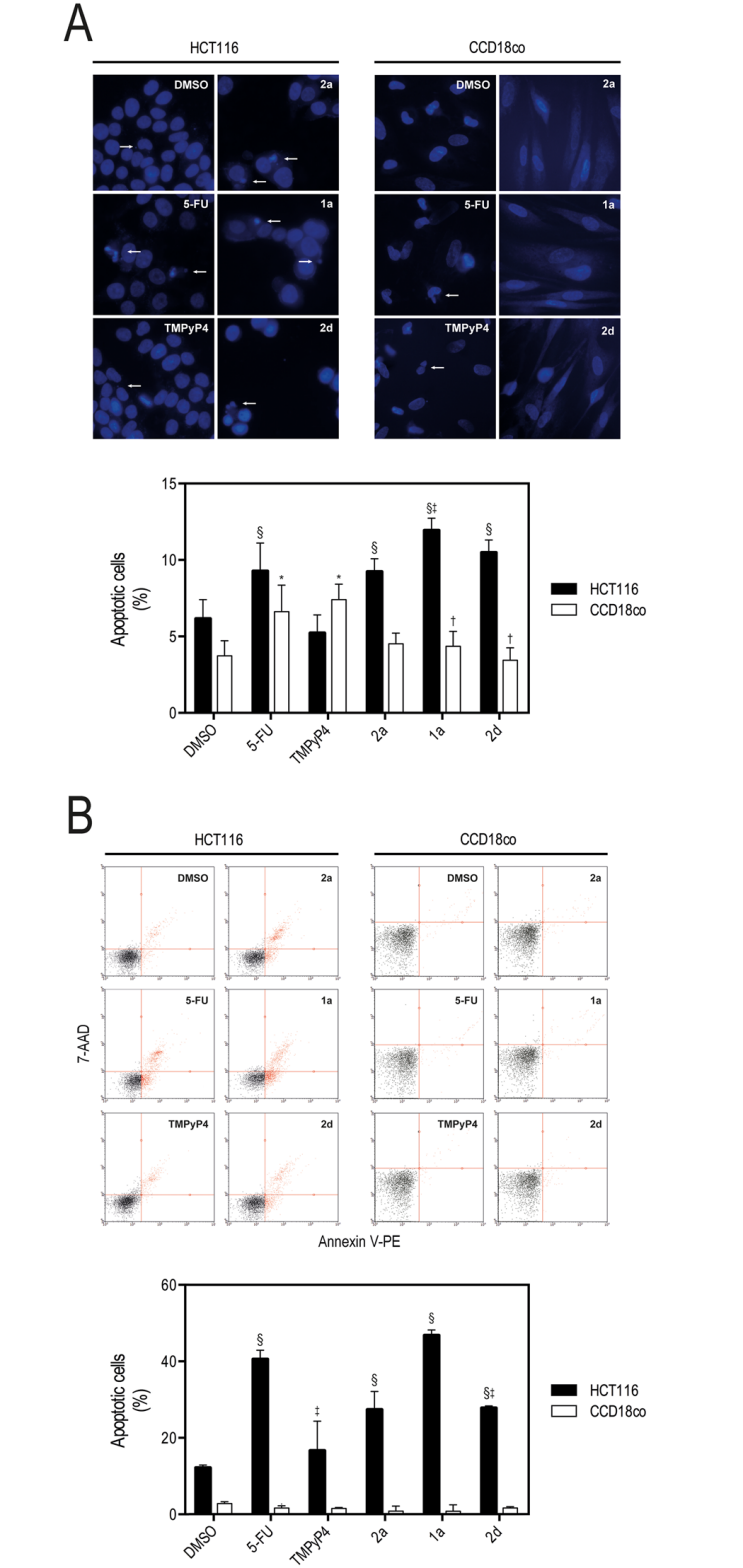
Exposure to IQ3A compounds increases apoptosis in HCT116 cells. **A.** Nuclear morphology and representative images of HCT116 human colon cancer cells and CCD18co human colon fibroblasts after Hoechst staining, evaluated by fluorescence microscopy after 72 h exposure to equitoxic (IC_50_) concentrations of 5-FU, TMPyP4 and IQ3A or DMSO (vehicle control) at 400x magnification. White Arrows indicate nuclear fragmentation and chromatin condensation, and **B.** Apoptotic cell populations obtained by Guava Nexin flow cytometry following 72 h incubation of HCT116 and CCD18co cells with 5-FU, TMPyP4 and IQ3A at equitoxic (IC_50_) concentrations, or DMSO (vehicle control). Results are expressed as the mean percentage (%) of early (LR quadrant) and late (UR quadrant) apoptotic cells. Results are expressed as mean ± SEM of at least three independent experiments; *p < 0.05 and §p < 0.01 from DMSO (vehicle control); and †p <0.05 and ‡p < 0.01 from 5-FU.

**Fig 5:**
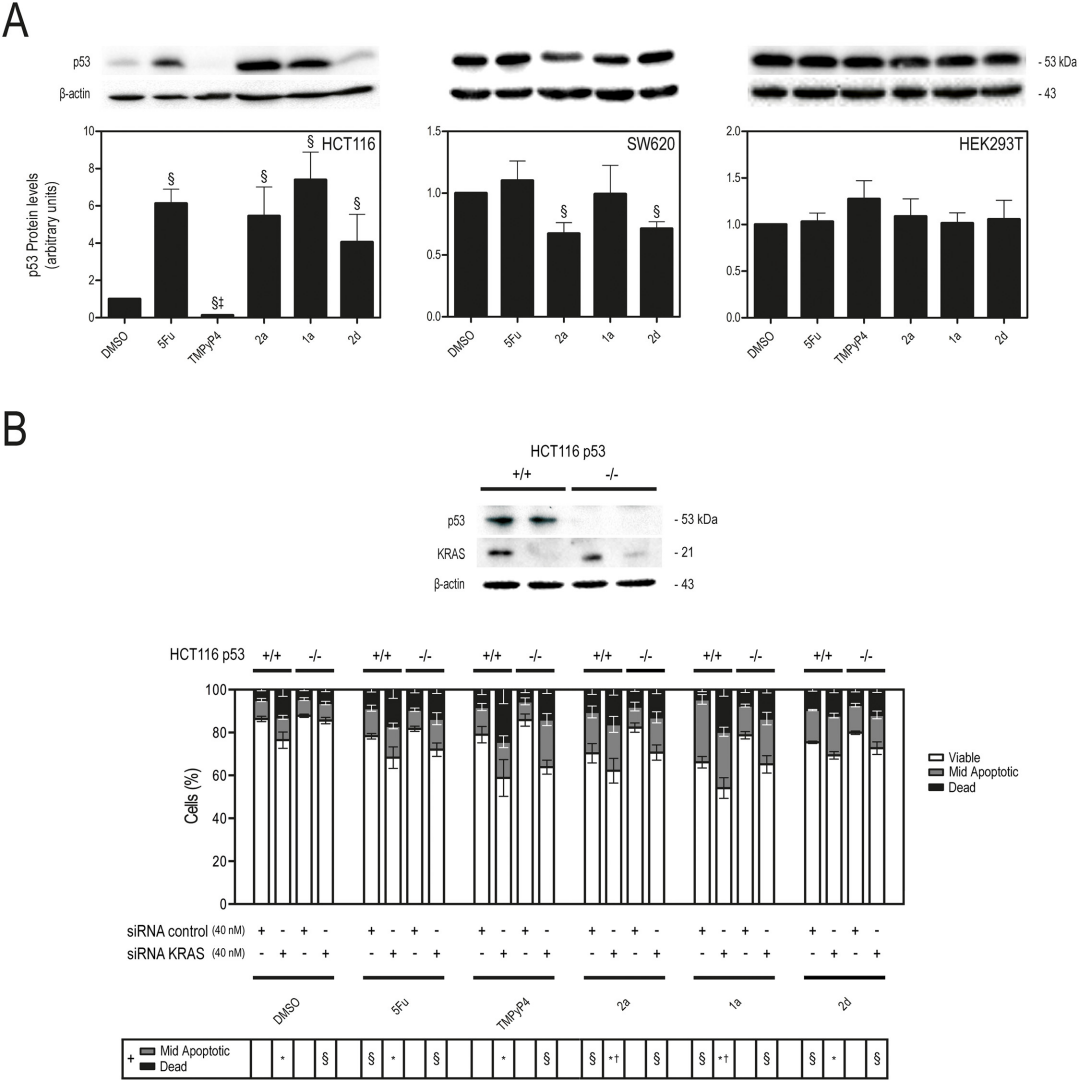
Exposure to IQ3A compounds increases p53 steady-state protein expression in HCT116 cells. **A.** p53 protein steady-state expression evaluated by immunoblot relative to DMSO (vehicle control), after 72 h exposure to equitoxic (IC_50_) concentrations of 5-FU, TMPyP4 and IQ3A. **B.** Cell populations obtained by Guava ViaCount flow cytometry following 72 h incubation of 5-FU, TMPyP4 and IQ3A at equitoxic (IC_50_) concentrations, or DMSO (vehicle control), in HCT116 p53 (+/+) and p53 (−/−) isogenic cell lines transfected with 40 nM siRNA KRAS or siRNA control. Results are expressed as mean ± SEM of at least three independent experiments. **A.** §p < 0.01 from DMSO (vehicle control); and ‡p < 0.01 from 5-FU. **B.** *p < 0.05 from siRNA control in P53 (+/+) cells; §p < 0.05 from siRNA control in p53 (-/-) cells; and †p <0.05 from siRNA KRAS in p53 (-/-) cells.

To further validate the effect of the IQ3A compounds on the induction of apoptosis in HCT116 cells, apoptosis was ascertained by evaluating changes in nuclear morphology by Hoechst staining, and also by the Nexin assay, following 72 h of incubation with IQ3A compounds at the IC_50_ concentrations. CCD18co cells were used in parallel to confirm that IQ3A do not elicit cell death in normal cells. These results showed that IQ3A significantly induce apoptosis in HCT116 cells, with compound **1a** producing a marked increase in apoptosis compared to vehicle (Fig 4A and 4B). In addition, we also confirmed that IQ3A compounds do not induce apoptosis in normal colon fibroblasts CCD18co. The p53 protein plays a central and pivotal role in human cancers [33], having a potent tumor suppressive activity via pleiotropic mechanisms. Therefore, the steady-state levels of p53 protein were evaluated by immunoblotting, following 72 h of incubation with IQ3A compounds at the IC_50_ concentrations, to ascertain their involvement on the mechanism of apoptosis elicited by IQ3A. Our data (Fig 5A) clearly demonstrate that IQ3A increased p53 protein steady state levels in HCT116 by 4–7 fold (*p* < 0.01) (Fig 5A, left panel), which may be correlated with the higher cell death verified by Guava ViaCount assay. In SW620 cells, no significant increase in p53 steady-state expression was detected, possibly due to the mutant status of p53 (Fig 5A, middle panel). Finally, in HEK293T cells, IQ3A showed no influence on p53 protein expression, in accord with the absence of cell death in the viability assay (Fig 5A, right panel). In addition, we further explored the relevance of p53 in the mechanism of apoptosis elicited by IQ3A, by silencing KRAS in HCT116 p53 wild-type (p53(+/+)) and null (p53(-/-)) isogenic cell lines, and evaluating its impact on cell death, and on IQ3A-induced cell death (Fig 5B). Our results clearly show that KRAS silencing induced a higher level of cell death compared to siRNA control (*p* < 0.05), and a higher level of IQ3A-induced cell death in HCT116 p53(+/+) compared to HCT116 p53(-/-) (*p* < 0.05 for **2a** and **1a**). Further, in siRNA control transfected cells, all IQ3A significantly induced a higher level of cell death in HCT116 p53(+/+) compared to HCT116 p53(-/-) (*p* < 0.05). However, in p53 mutant SW620 cells, IQ3A compounds did not significantly elicit cell death (Fig 3), nor did they increase p53 protein steady-state expression (Fig 5). In agreement, KRAS and/or HSP90 silencing were unable to induce cell death in this cell line, whereas KRAS silencing induced a dose-dependent effect in the reduction of SW620 cell proliferation (S3 Fig). Collectively, these data further confirm the importance of p53 for IQ3A induction of apoptosis.

**Fig 6:**
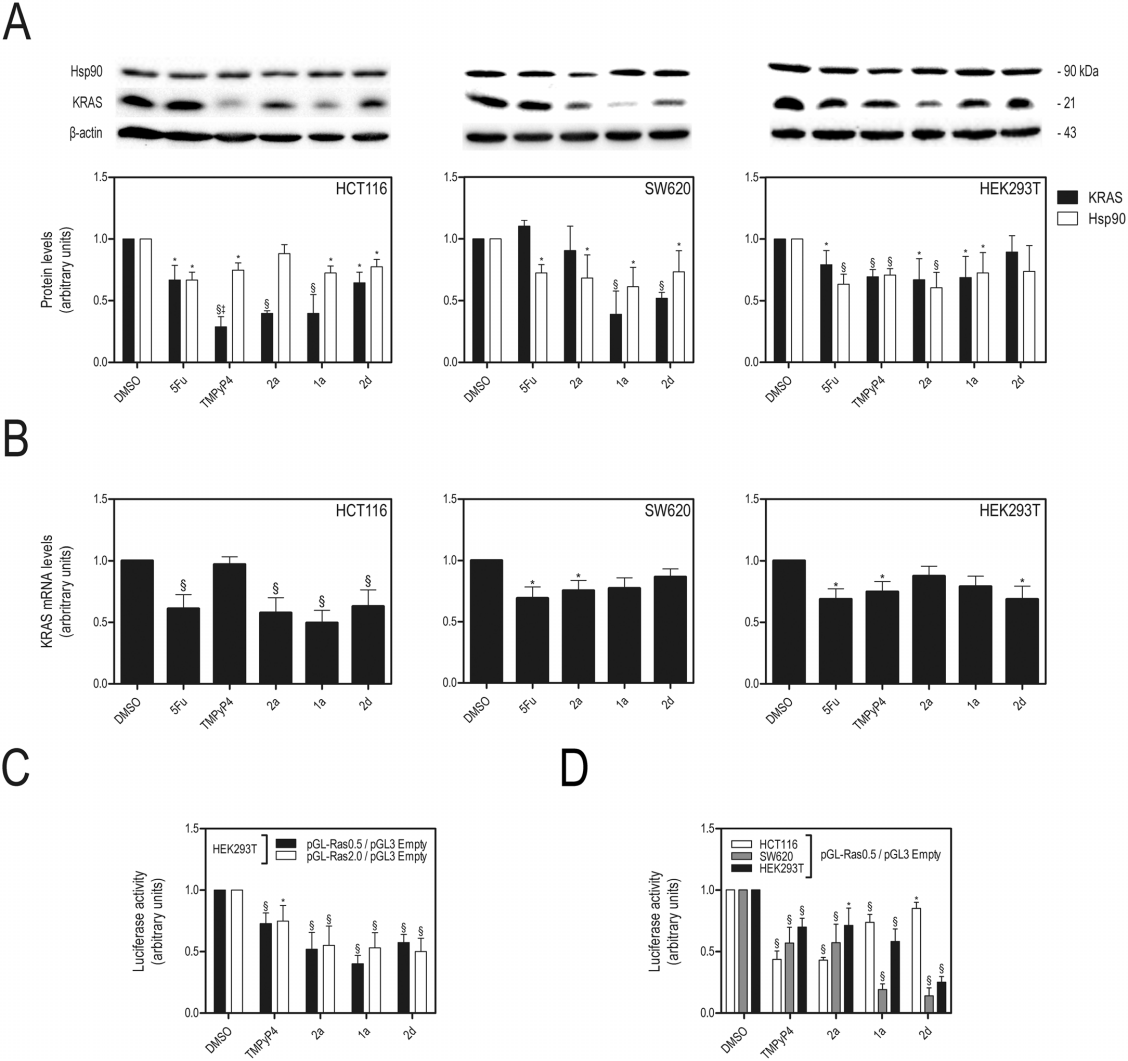
Exposure to IQ3A compounds decreases *HSP90* and *KRAS* mRNA, protein expression and *KRAS* transcription. **A.** KRAS and HSP90 protein steady-state expression evaluated by immunoblot relative to DMSO (vehicle control), after 72 h exposure to equitoxic (IC_50_) concentrations of 5-FU, TMPyP4 and IQ3A treatment; **B.** *KRAS* mRNA steady-state expression was evaluated by Taqman Real-time RT-PCR using specific Taqman Assays for KRAS and β-Actin for normalization. *KRAS* mRNA steady-state expression levels were calculated by the ΔΔCt method, using DMSO (vehicle control) for calibration; and **C.** HEK293T cells were co-transfected with pGL3-basic vector (empty vector control), or with *KRAS* promoter luciferase reporter construct PGL-Ras0.5, or PGL-Ras2.0, together with pRL-TK. Twenty-four hours later, cells were replated in 96-well plates, at 5.000 cells per well. Subsequently, 24 h after replating, cells were exposed to IC_50_ equitoxic concentration of test compounds IQ3A, TMPyP4 and vehicle (DMSO); **D.** HCT116, SW620 and HEK293T cells were co-transfected with pGL3-basic vector (empty vector control), or with KRAS promoter luciferase reporter construct PGL-Ras0.5, together with pRL-TK. Twenty-four hours later, cells were replated in 96-well plates, at 5,000 cells per well and exposed to IC_50_ equitoxic concentration of test compounds IQ3A, TMPyP4 and vehicle (DMSO). *KRAS* promoter activity levels were evaluated by Dual-Luciferase assay 72 h (**C.**) or 24 h (**D.**) after compound exposure. Results are expressed as the luciferase signal ratio of pGL-Ras2.0 or pGL-Ras0.5 to pGL3-basic vector transfected cells, after normalization with Renilla Luciferase. Results are expressed as mean ± SEM of at least three independent experiments; *p < 0.05 and §p < 0.01 from DMSO (vehicle control); and †p <0.05 and ‡p < 0.01 from 5-FU.

Finally, the capacity of IQ3A compounds to repress *KRAS* expression was evaluated by quantifying KRAS protein in cancer cell lines. Since IQ3As have also shown to be effective stabilizers of G4 sequences in the HSP90 oncogene promoter region (Table 1), the expression of this protein was also evaluated. Fig 6A shows that G4 ligands **1a**, **2a**, **2d** and TMPyP4 down-regulated the expression of mutant KRAS by 35–60% in HCT116 and SW620 cells, with exception of **2a** (*p* < 0.01). In addition, IQ3A also reduced HSP90 protein steady levels, but to a lesser extent compared to KRAS. Therefore, we next investigated the ability of IQ3A compounds to down-regulate *KRAS* transcription in colon cancer cells. *KRAS* mRNA steady-state levels were evaluated by RT-PCR after 72 h incubation of cells with the compounds at their IC_50_ concentrations and compared with the effect of TMPyP4 at equitoxic concentrations. G4 ligands such as TMPyP4 have been shown to bind to G4 structures from the *KRAS* promoter region and from the 5’-UTR of *KRAS* mRNA, and also repress both gene transcription and translation [13,19]. Fig 6B shows that **1a**, **2a** and **2d** were able to down-regulate KRAS transcription by ca 40% in HCT116 cells, but not significantly so in SW620 cells. A possible explanation is the time point at which we evaluated *KRAS* mRNA and protein steady-state levels. After 72 h of IQ3A exposure, there may no longer be significant repression of *KRAS* promoter activity in the SW620 cell line, whereas protein levels remain reduced as a result of accumulated IQ3A effects. Also, TMPyP4 was unable to reduce *KRAS* mRNA steady-state levels in the HCT116 cell line, in contrast to a decrease of ca 80% of the protein steady-state levels (Fig 6A and 6B). These results are in agreement with the reported ability of TMPyP4 to preferentially accumulate in the cytoplasm of cells [19], where it can inhibit *KRAS* mRNA translation.

To validate that the mechanism of anti-proliferative activity and apoptotic induction by IQ3A compounds involves repression of *KRAS* gene expression as a result of the stabilization of G4-forming sequences present in the promoter, the effects of compounds on the *KRAS* gene promoter were directly evaluated by a luciferase reporter assay. For this purpose, we used two different size promoter constructs containing the G4 region of the *KRAS* gene promoter, cloned into the pGL3 Basic backbone: pGL-Ras0.5, pGL-Ras2.0, and pGL3 Basic empty (Firefly Luciferase negative control/no promoter), co-transfected together with pRL-TK (transfection efficiency normalization) into HEK293T cells, as a G4 negative control. This construct does not harbor G4 sequences and is insensitive to G4-related effects/regulation. Our data clearly demonstrate that IQ3A compounds, similarly to TMPyP4, were able to significantly reduce *KRAS* transcription, we suggest by interacting with the G4 region of the *KRAS* gene promoter, suppressing downstream coding-region expression from 40 to 60% versus DMSO control (*p* < 0.01) (Fig 6C). Using both plasmids, with 500 and 2000 bp upstream to the transcription start site, we also show that the target region of the IQ3A compounds is within this region, thus coinciding with the polypurine G-rich strand responsible for G-quadruplex structure assembly [12]. Importantly, we were also able to show that IQ3A compounds, similarly to TMPyP4, were able to significantly reduce *KRAS* promoter activity in HCT116 and SW620 cells (Fig 6D).

### Conclusions

We have investigated in this study the ability of a group of 7-carboxylate indolo[3,2-*b*]quinoline tri-alkylamine derivatives (IQ3A), which are potent stabilizers of DNA G4 structures present in the *KRAS* promoter, to down-regulate *KRAS* expression and induce cell death by apoptosis, particularly in KRAS-dependent colon cancer cell lines. The IQ3A compounds markedly showed anti-proliferative activity in an IC_50_ range lower than 2.69 μM in HCT116 and SW620 cells. Particularly, in HCT116 cells, IQ3A compounds at the IC_65_ concentration increased cell death up to 4.7 fold (*p* < 0.01) and 2.4 fold (*p* < 0.01) in comparison to DMSO or 5-FU, respectively. In addition, in non-malignant cell lines the IQ3A compounds did not cause significant cell death. Further, they markedly reduced *KRAS* mRNA expression and protein levels in colon cancer cells possibly through direct transcriptional repression of the *KRAS* promoter. Our data to date shows a correlative relationship between transcriptional repression and binding to the G4 region within this promoter, although further studies are needed in order to demonstrate a direct link between the two. The repression effect was accompanied by increased p53 protein steady-state levels in HCT116, and only a slight reduction of HSP90 levels in the three cell lines tested, indicating that these compounds are not also having an effect on some other possible G4 targets such as in the HSP90 promoter [27]. Rather the IQ3A compounds are selectively targeting the *KRAS* driver genes in the colon cancer cell lines. Collectively these results show that G4-binding ligands, such as these indoloquinoline derivatives, display potential to be employed as novel anticancer therapeutic agents, especially for the treatment of human cancers driven by KRAS mutations, which are still in large part cancers of high unmet clinical need.

## Materials and Methods

### Chemicals

7-carboxylate indolo[3,2-*b*]quinoline tri-alkylamine derivatives **1a-d** and **2a-d** (IQ3A) were synthesized as described in S1 Text. 5-fluorouracil (5-FU) and TMPyP4 were purchased from Sigma-Aldrich, St Louis, MO, USA.

### Oligonucleotides sequences

All oligonucleotides were purchased from Eurofins MWG Synthesis GmbH, Germany. The labeled oligonucleotides used in the FRET assays had attached the donor fluorophore FAM (6-carboxyfluorescein) and the acceptor fluorophore TAMRA (6-carboxytetramethylrhodamine): KRAS21R (5’-FAM-AGGGCGGTGTGGGAAGAGGGA-TAMRA-3’); HSP90A (5’- [FAM]-GGGCCAAAGGGAAGGGGTGGG-[TAMRA]-3’); T-Loop (5’-FAM-TATAGCTATATTTTTTTATAGCTATA-TAMRA-3’). Each oligonucleotide was initially diluted to a storage solution at 100 μM in nuclease free water (not DEPC-treated), purchased from Ambion Applied Biosystems UK.

### FRET melting assay

The ability of IQ3A and TMPyP4 to stabilize G-quadruplex and duplex DNA sequences was investigated using a Fluorescence Resonance Energy Transfer (FRET) assay. Stock solution of the oligonucleotides sequences at 20 μM and subsequent dilutions were obtained from storage solutions diluting with K-cacodylate buffer pH = 7.4, containing 60 mM K^+^ (10 mM potassium cacodylate, 50 mM KCl). The FRET probe sequences were diluted from stock to the correct concentration (0.4 μM) and then annealed by heating to 95°C for 10 min, followed by cooling to RT. Test compounds were prepared as 1 mM in 10% DMSO and 10% 1mM HCl in HPLC-grade water. The rest of the dilutions were performed using FRET buffer. Annealed DNA (50 μL) and compound solution (50 μL) were distributed across 96-well RT-PCR plates (BioRad; MJ Research, Waltham, MA, USA). Relevant controls were also performed to check for interference with the assay. Fluorescence readings were made on a DNA Engine Opticon (MJ Research) with excitation at 450–495 nm and detection at 515–545 nm, taken at intervals of 0.5°C in the range 30–100°C, with a constant temperature being maintained for 30 s prior to each reading to ensure a stable value. Experiments were performed in triplicate. Final analysis of the data was carried out with GraphPad Prism 5.0 software (GraphPad Software, Inc). The advanced curve-fitting function in GraphPad Prism was used for calculation of Δ*T*_*m*_ values and associated standard deviations.

### Cell culture

SW620 human colorectal adenocarcinoma cells (European Collection of Cell Cultures, ECACC, Porton Down, Wiltshire, UK), HEK293T human embryonic kidney cells (ATCC, LGC Standards, UK) HCT116 human colon carcinoma cells (ECACC), HCT116 p53(+/+) and p53(−/−) isogenic human colon cancer cells (GRCF Cell Center and Biorepository, the Johns Hopkins University, School of Medicine, Baltimore, MD, USA), were grown in Dulbecco’s modified Eagle’s medium (DMEM) supplemented with 10% fetal bovine serum, and 1% antibiotic/antimycotic (Invitrogen, Grand Island, NY, USA), CCD18co human colon fibroblasts (ATCC, CRL-1459) were grown in Minimum Essential Medium (MEM) supplemented with 10% fetal bovine serum, 1% antibiotic/antimycotic, 2 mM GlutaMAX, 0,1 mM Non-essential amino acids (NEAA) (Invitrogen) and 0.57 mM Recombinant Human TNF-α (Peprotech, USA) and maintained at 37°C in a humidified atmosphere of 5% CO_2_.

### Evaluation of cell viability and general cell death

Cell viability was evaluated by the tetrazolium dye (MTS) Short-Term Cytotoxicity Assay. In brief, cells were seeded in 96 well plates at 5,000 cells/well. Twenty-four hours after cell plating, media was removed and replaced with fresh media containing test compounds IQ3A, TMPyP4 and 5-FU, or vehicle control (DMSO). Following 72 h of compound exposure, cell viability was evaluated using the CellTiter 96 AQueous Non-Radioactive Cell Proliferation Assay (Promega, Madison, WI, USA), using 3-(4,5-dimethylthiazol-2-yl)-5-(3-carboxymethoxyphenyl)-2-(4-sulfophenyl)-2H-tetrazolium, inner salt (MTS) as previously described [34] [35]. Cell viability data were expressed as mean ±SD from at least three independent experiments. IC_50_ and IC_65_ values were determined using GaphPad Prism v.5.00 (GraphPad Software). In selected experiments, cell viability was also assessed by trypan blue exclusion assay [34], and general cell death by LDH activity from cell culture supernatants [7].

### Guava ViaCount assay

ViaCount assay was used with Guava easyCyte 5HT Flow cytometer (Guava Technologies, Inc., Hayward, CA, USA), as previously described [36] to evaluate viable, apoptotic and dead cell populations, on HCT116, SW620, HEK293T and CCD18co cells exposed to test compounds IQ3A, TMPyP4 and 5-FU at IC_50_ and IC_65_ concentrations, and a vehicle control (DMSO). The ViaCount Assay distinguishes viable mid apoptotic and dead cells based on differential permeability of two DNA-binding dyes in the GuavaVia Count Reagent. The nuclear dye stains only nucleated cells, while the viability dye brightly stains dying cells. HCT116, SW620, HEK293 T and CCD18co cells were seeded in 24-well plates 50,000 cells/well. Twenty-four hours later, cells were exposed to compounds for 72 h. After treatment, cell culture supernatants were collected and adherent cells were detached with TrypLE (Invitrogen). Next, detached cells were pooled with cell culture supernatants and centrifuged for 5 min (650 g). Supernatants were discarded and the cells were resuspended in 50–500μl phosphate buffered saline (PBS) with 2% FBS. Subsequently, 15 μl of cell suspension were mixed with 135 μl of Guava ViaCount reagent, and incubated for 5 min at room temperature. Sample acquisition and data analysis were performed using the ViaCount software module.

### Guava Nexin assay

Nexin assay was used with Guava easyCyte 5HT Flow cytometer (Guava Technologies, Inc., Hayward, CA, USA) to evaluate viable, early and late apoptotic cell populations, on HCT116 and CCD18co cells exposed to either test compounds IQ3A, TMPyP4 and 5-FU at IC_50_ concentration, or vehicle control (DMSO). The Nexin Assay distinguishes viable, early and late apoptotic cells based on the externalization of phosphatidylserine to the cell surface, where Annexin V can readily bind them. The membrane dye stains with higher intensity early and late apoptotic cells. HCT116 and CCD18co cells were seeded in 24-well plates 50,000 cells/well. Twenty-four hours later, cells were exposed to compounds for 72 h. After treatment, cell culture supernatants were collected and adherent cells were detached with TrypLE (Invitrogen). Next, detached cells were pooled with cell culture supernatants and centrifuged for 5 min (650 g). Supernatants were discarded and the cells were resuspended in 50–500 μl phosphate buffered saline (PBS) with 2% FBS. Subsequently, 50 μl of cell suspension were mixed with 50 μl of Guava Nexin reagent, and incubated for 20 min at room temperature. Sample acquisition and data analysis were performed using the Nexin software module.

### Total protein extraction and immunoblotting

HCT116, SW620 and HEK293 T cells were seeded in 35 mm plates at 300,000 cells per well. Test compounds IQ3A, TMPyP4, 5-FU and a vehicle (DMSO) were added to the cells 24 h after plating, at IC_50_ equitoxic concentration. After 72 h of compound exposure, cells were collected and processed for total protein extraction, as previously described [8]. Briefly, samples were homogenized in ice-cold 1:1 solution of buffer A [10 mM Tris•HCl pH 7.6, 5 mM MgCl_2_, 1.5 mM KAcO, 2 mM dithiothreitol (DTT), and Halt Protease and Phosphatase inhibitor cocktail, EDTA-free (#78445, Thermo Scientific)] and buffer 2X (10 mM Tris•HCl pH 7.6, 1% Nonidet-P40, and Halt Protease and Phosphatase inhibitor cocktail), by vigorous vortexing and incubated on ice for 30 min. Samples were then sonicated (two cycles of 15 s sonication and 30 s ice incubation, using a compact ultrasonic device with amplitude adjusted to 80% and pulse to 90%; model UP100H, Hielscher Ultrasonics GmbH, Teltow (Germany); 100 W, ultrasonic frequency: 30 kHz) and centrifuged at 10,000 g for 10 min at 4°C. The clear supernatants containing the total protein extracts were transferred to a fresh tube and stored at −80°C. Protein concentrations were determined using the BioRad protein assay kit according to the manufacturer’s instructions. Steady-state levels of KRAS and Hsp90 protein were determined by immunoblot analysis. Briefly, 50–100 μg of total protein extracts were separated by 12% SDS-PAGE. After electrophoretic transfer onto nitrocellulose membranes, immunoblots were blocked with 5% milk solution, and next incubated overnight at 4°C with primary mouse monoclonal antibody reactive to KRAS (#sc-30; Santa Cruz Biotechnology Inc., Santa Cruz, CA, USA) and to Hsp90 (#sc-13119; Santa Cruz Biotechnology Inc., Santa Cruz, CA, USA). Finally, immunoblots were incubated with secondary anti-mouse antibody conjugated with horseradish peroxidase (BioRad) for 3 h at room temperature. The membranes were processed for protein detection using Super Signal substrate (Pierce, Rockford, IL, USA). β-Actin (#A-5441, Sigma—Aldrich) was used as a loading control. Steady-state protein levels were expressed as mean ±SEM from at least three independent experiments.

### Total RNA extraction and Taqman Real-time RT-PCR

HCT116, SW620 and HEK293 T cells were seeded in 35 mm plates at 300,000 cells per well. Test compounds IQ3A, TMPyP4, and 5-FU and vehicle (DMSO) were added to the cells 24 h after plating, at IC_50_ equitoxic concentration. After 72 h of compound exposure, cells were collected and processed for total RNA extraction using TRIZOL reagent (Invitrogen) according to the manufacturer’s instructions. Samples were homogenized in TRIZOL reagent using a motor-driven Bio-vortexer (No1083; Biospec Products, Bartlesfield, OK) and disposable RNAse/DNAse free sterile pestles (Thermo Fisher Scientific, Inc., Chicago, IL). RNA was quantified using a NanoDropH spectrophotometer, and typically showed A260/280 ratios between 1.9 and 2.1. Evaluation of steady-state expression of *KRAS* mRNA was performed by Taqman Real-time PCR. In brief, RT reactions were performed using 1.5 μg of total RNA, using High Capacity RNA to cDNA kit, according to the manufacturer`s instruction. Next, Real-time PCR reactions were performed using Taqman Universal Master Mix II, no UNG, and primers specific to human *KRAS* (assay ID Hs00000174_rf, # 4465807) primers and to human β-Actin (ACTB # 401846) for normalization to endogenous control (both from Applied Biosystems Inc). Triplicate reactions were run per sample. Data was collected with 7300 System Sequence Detection Software, version 1.2.3 (Applied Biosystems Inc). The comparative threshold cycle method was used to calculate the amplification factor, where the threshold cycle (Ct) is defined as the cycle number at which the fluorescence passes the fixed threshold intensity level. KRAS expression levels in different samples were calculated on the basis of ΔΔCt method. For each cell line, vehicle control (DMSO) was used as the calibrator. The n-fold change in KRAS expression was obtained using the formula: 2^-ΔΔCt^.

### KRAS and Hsp90 silencing

HCT116 p53 (+/+) and p53 (−/−) cells were seeded in 35 mm plates at 300,000 cells per well. Twenty-four hours later, cells were transfected with 80 nM KRAS *Silencer* Select Pre-Designed & Validated siRNA (siRNA KRAS) or 80 nM Silencer Negative Control siRNA (siRNA control) (both from Applied Biosystems, Foster City, CA, USA). In parallel SW620 cells were seeded in 35 mm plates at 300,000 cells per well. Twenty four hours later, cells were transfected with 50 or 100 nM siRNA KRAS, siRNA control, siRNA HSP90 (HSP90α/β siRNA (h) (sc-35608 Santa Cruz Biotechnology Inc., Santa Cruz, CA, USA), and co-transfected with 50 nM siRNA KRAS plus 50 nM siRNA HSP90. Transfections were performed using lipofectamine 3000 (Invitrogen), according to manufacturer’s instructions. Twenty-four h later, cells were replated in 24-well plates at 50,000 cells/well and 72 h later Guava ViaCount, trypan blue dye exllusion, MTS metabolism and LDH release assays.

### KRAS promoter activity Luciferase Reporter Assay

HEK293T cells were seeded in 35 mm plates at 300,000 cells per well. Twenty-four h later, cells were transiently co-transfected with pGL3-basic vector (empty vector control), or with KRAS promoter luciferase reporter construct PGL-Ras0.5, or PGL-Ras2.0, together with pRL-TK (Promega, Madison, WI, USA). KRAS promotor luciferase reporters respectively harbor 500 bp and 2000 bp of the human KRAS promotor region, kindly provided by Prof. Kim Nam-Soon. pGL3 Basic empty was used as negative control, and pRL-TK simultaneously for transfection efficiency normalization and as a G4 negative control. This construct does not harbor G4 sequences, therefore being insensitive to G-quadruplex-related effects/regulation. Transfections were performed using Lipofectamine 3000 (Invitrogen), according to the manufacturer's instructions. Twenty-four h after transfection, cells were replated in 96-well plates, at 5,000 cells per well. Subsequently, 24 h after replating, test compounds IQ3A, TMPyP4, and vehicle (DMSO) were added to the cells at IC_50_ equitoxic concentration. Finally, 72 h after compound incubation, cells were lysed and firefly and renilla luciferase activities were measured using Dual-Luciferase Reporter Assay System (Promega). KRAS promoter activity levels were expressed as the luciferase signal ratio of pGL-Ras2.0 or pGL-Ras0.5 to pGL3-basic vector transfected cells, after normalization with Renilla Luciferase. In parallel, HCT116, SW620 and HEK293T cells were transfected with PGL-Ras0.5 together with pRL-TK (Promega, Madison, WI, USA), replated in 96-well plates and simultaneously exposed to test compounds IQ3A, TMPyP4, and vehicle (DMSO) at IC_50_ equitoxic concentration. Twenty-four h later, cells were lysed and renilla luciferase activities were measured and expressed as above. The results are expressed as the mean ± SEM fold-change compared to DMSO exposure, from three independent experiments.

## Supporting Information

S1 Fig HPLC-ELSD chromatograms and MS spectra of 2a and 2d. (PDF)

S2 Fig FRET melting profiles of 1a-d and 2a,b,d. (PDF)

S3 Fig Effect of KRAS and/or HSP90 silencing in SW620 cells. SW620 cells were transfected with 50 or 100 nM siRNA KRAS, siRNA HSP90 or siRNA control, and co-transfected with 50 nM siRNA KRAS plus 50 nM siRNA HSP90. Twenty-four h later, cells were replated in 24 well plates at 50,000 cells/well, and 72 h later (96 h of transfection) cells were processed for: **A.** Evaluation of steady-state expression of KRAS and HSP90 protein by immunoblot; **B.** Guava ViaCount assay (upper panel) and trypan blue exclusion assay (lower panel); **C.** LDH release assay (upper panel) and MTS metabolism Assay (lower panel). Results are expressed as mean ± SEM of three independent experiments; §p < 0.01 from siRNA control (50 nM); and ‡p < 0.01 from siRNA control (100 nM).
(TIF)

S1 Text Synthetic procedure of the 7-carboxylate indolo[3,2-*b*]quinoline tri-alkylamine derivatives (IQ3A). (PDF)
