# Supplementary material for: Targeting KRAS Oncogene in Colon Cancer Cells with 7-Carboxylate Indolo[3,2-b]quinoline Tri-Alkylamine Derivatives
Source: PLoS One. 2015 May 29;10(5):e0126891. doi: 10.1371/journal.pone.0126891 (PMC4449006; doi:10.1371/journal.pone.0126891)
Supplement: S1 Fig — (PDF) [file pone.0126891.s001.pdf]

HPLC-ELSD-MS analysis

Purity was determined by analytical LC-ELSD-MS using 1mg/ml solutions of the compounds in DMSO. The analysis was performed on a Waters 2545 HPLC equipped with a 2998 UV-Vis diode array detector, a Waters 3100 ESI-MS module and a Waters 2424 ELS Detector, using a XTerraMS C18 5 µm, 4.6 × 50 mm column at a flow rate of 5 mL/min with a linear gradient (95%A:5%B to 100%B with 90 and 30s hold at 100% B, solvent A = water + 0.1% formic acid, solvent B = acetonitrile + 0.1% formic acid).

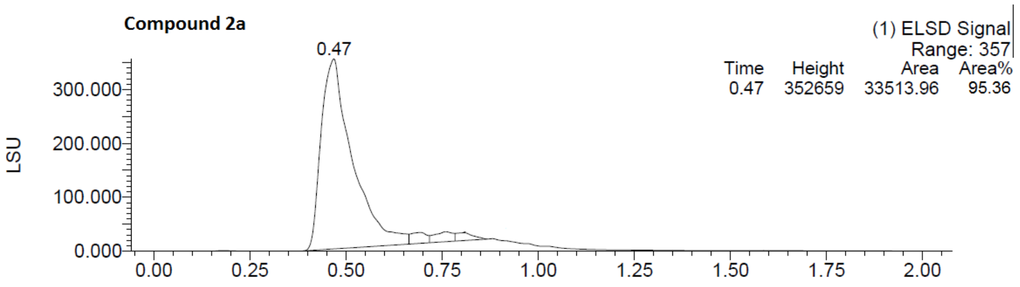

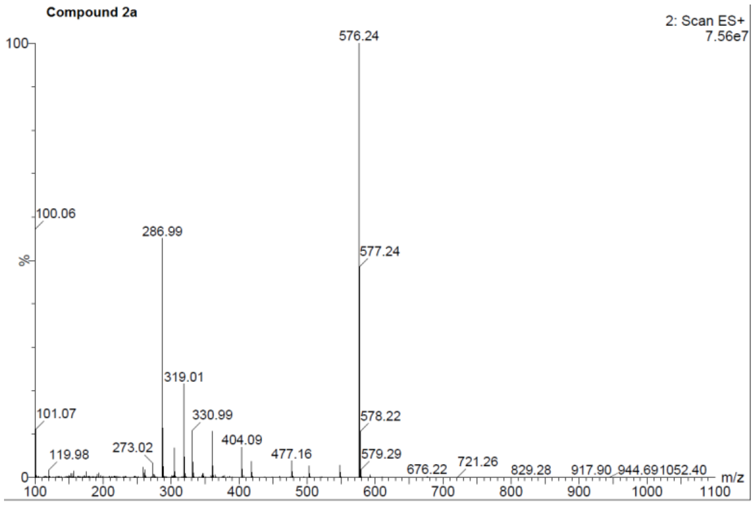

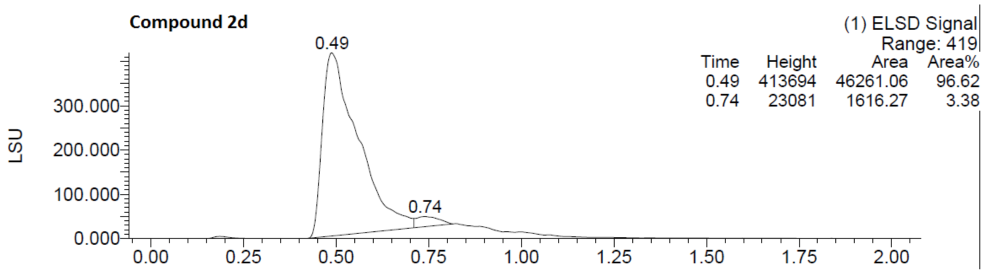

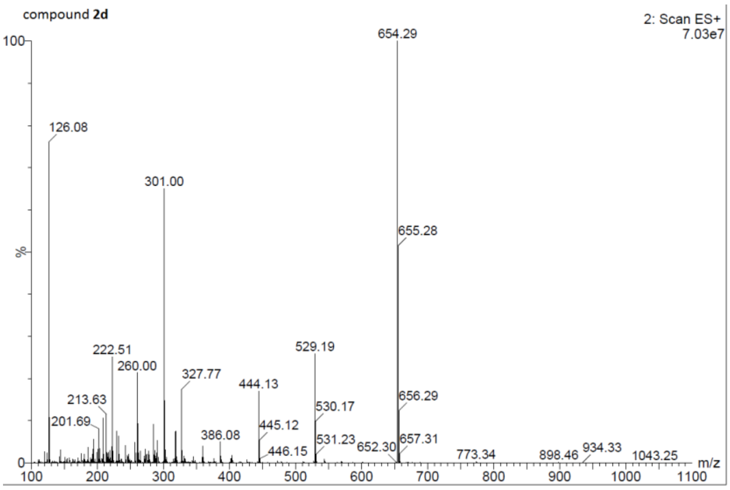

Figure S2 – HPLC-ELSD chromatograms and MS spectra of **2a** and **2d**
