# Supplementary figures and images for: Targeting KRAS Oncogene in Colon Cancer Cells with 7-Carboxylate Indolo[3,2-b]quinoline Tri-Alkylamine Derivatives
Source: PLoS One. 2015 May 29;10(5):e0126891. doi: 10.1371/journal.pone.0126891 (PMC4449006; doi:10.1371/journal.pone.0126891)

a)

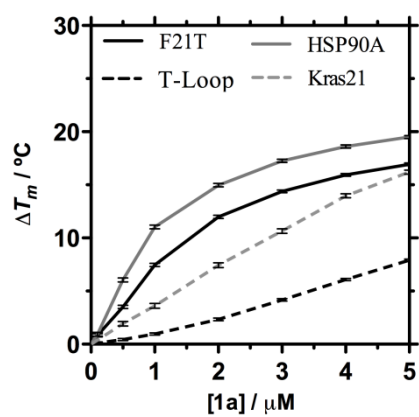

b)

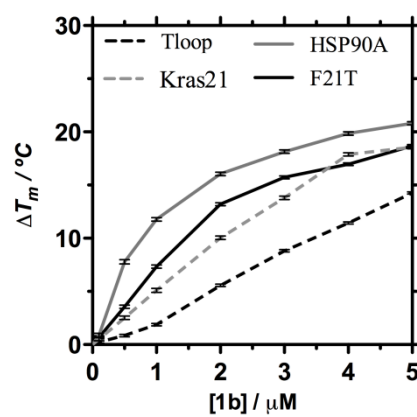

c)

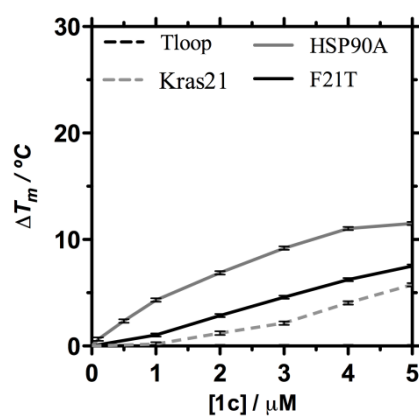

d)

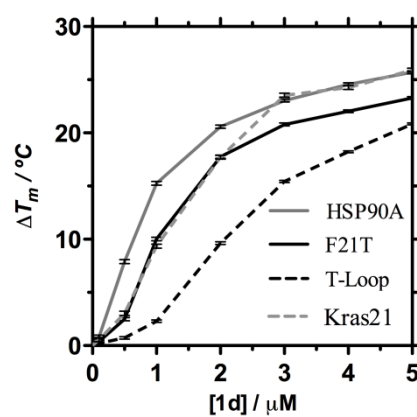

**Figure S3A.** FRET melting profiles of **1a-d**.

a)

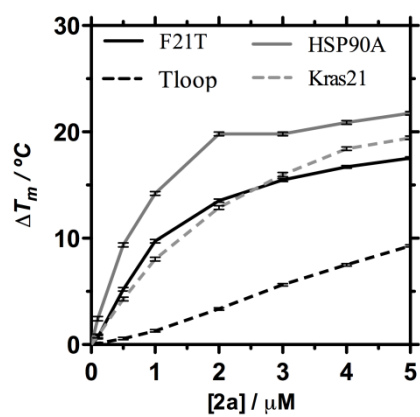

b)

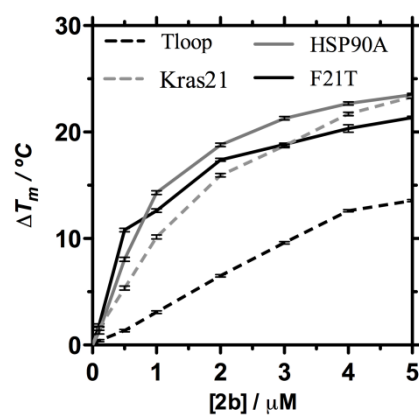

c)

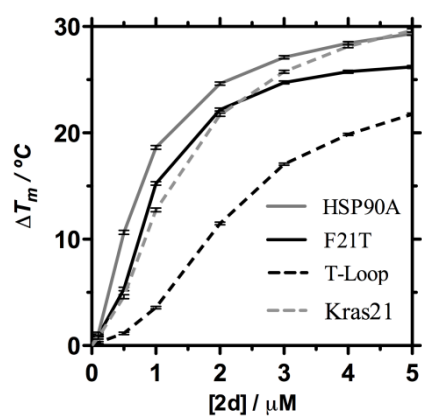

**Figure S3B.** FRET melting profiles of **2a,b,d**.

Supplement: S2 Fig — (PDF) [file pone.0126891.s002.pdf]

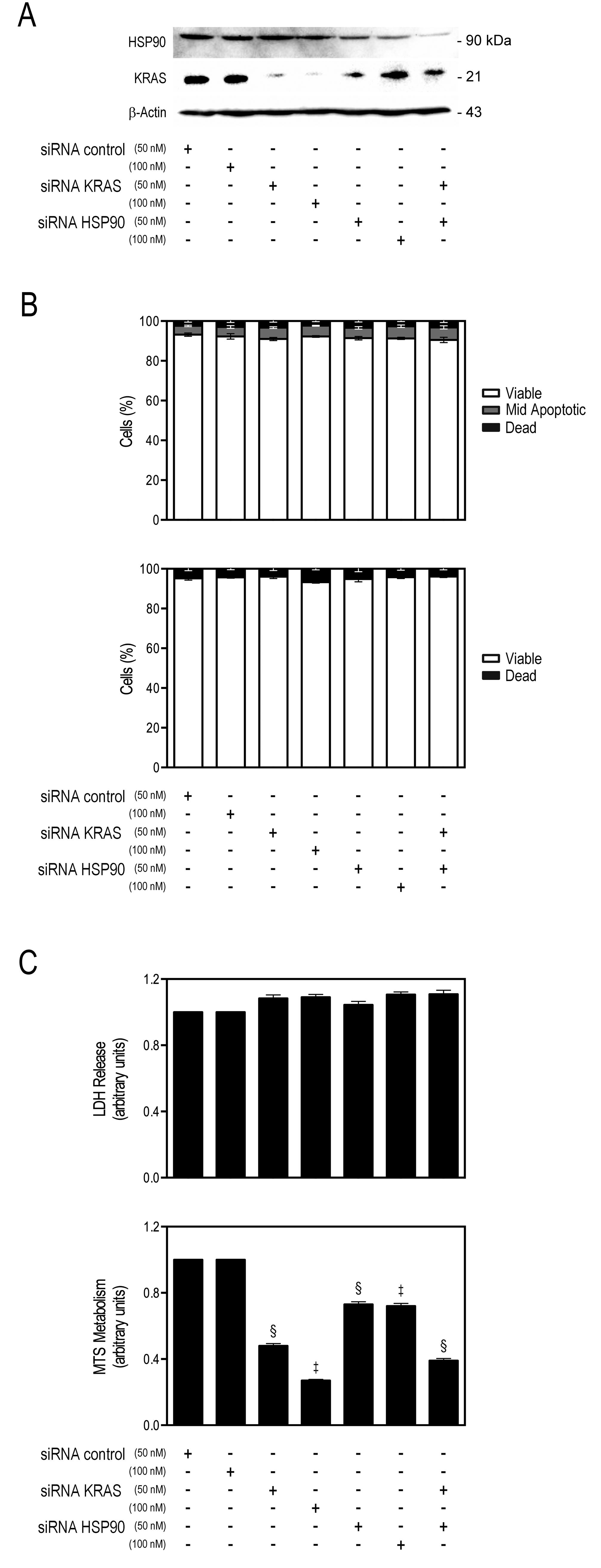

Supplement: S3 Fig — SW620 cells were transfected with 50 or 100 nM siRNA KRAS, siRNA HSP90 or siRNA control, and co-transfected with 50 nM siRNA KRAS plus 50 nM siRNA HSP90. Twenty-four h later, cells were replated in 24 well plates at 50,000 cells/well, and 72 h later (96 h of transfection) cells were processed for: A. Evaluation of steady-state expression of KRAS and HSP90 protein by immunoblot; B. Guava ViaCount assay (upper panel) and trypan blue exclusion assay (lower panel); C. LDH release assay (upper panel) and MTS metabolism Assay (lower panel). Results are expressed as mean ± SEM of three independent experiments; §p < 0.01 from siRNA control (50 nM); and ‡p < 0.01 from siRNA control (100 nM). (TIF) [file pone.0126891.s003.tif]
