# Supplementary material for: Targeting KRAS Oncogene in Colon Cancer Cells with 7-Carboxylate Indolo[3,2-b]quinoline Tri-Alkylamine Derivatives
Source: PLoS One. 2015 May 29;10(5):e0126891. doi: 10.1371/journal.pone.0126891 (PMC4449006; doi:10.1371/journal.pone.0126891)
Supplement: S1 Text — (PDF) [file pone.0126891.s004.pdf]

Synthesis: General Methods

Chemicals were purchased from Sigma-Aldrich Chemical Co. Ltd., Spain and used without further purification. Microwave reactions were made on a CEM Focused Microwave™ Synthesis System, Model Discover, equipped with the IntelliVent Pressure Control System. All compounds were characterized by NMR spectroscopy, recorded on a Bruker Avance 400 spectrometer at 400 MHz (^{1}H NMR) and 100 MHz (^{13}C NMR) and using solvent as internal reference. Chemical shifts (δ) are expressed in ppm. Signal splitting patterns are described as singlet (s), doublet (d), triplet (t), quartet (q), quintet (quint.), multiplet (m), broad signal (br) or combination thereof. Coupling constants (*J*) are given in Hz. The purity of compounds submitted to biological tests were in all cases ≥ 95% as determined by HPLC-ELSD-MS. Melting points (mp) were determined using a Bock-Monoscop M. Reactions were monitored by thin-layer chromatography (TLC) using coated silica gel plates (Merck, aluminium sheets, silica gel 60 F254) and aluminium oxide matrix plates (Sigma-Aldrich, PET support, F254). Preparative thin layer chromatography (PTLC) was performed in neutral aluminium oxide 60 G type E (Merck, 200x200 mm glass support).

Synthetic procedure

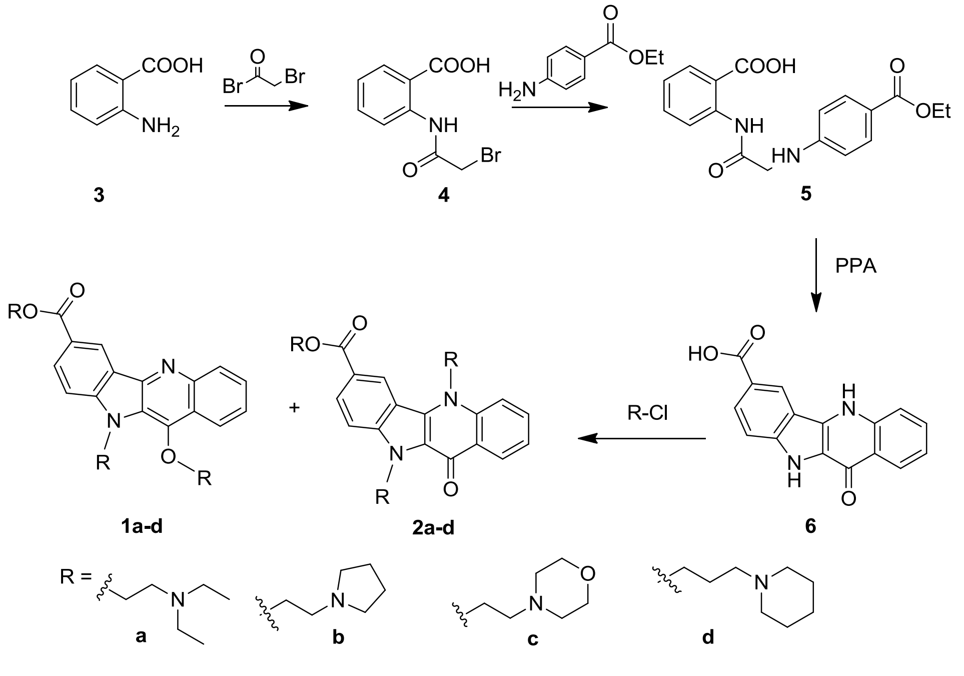

Scheme. Synthetic procedure of the tri-alkylamine 7-carboxylate indolo[3,2-*b*]quinolines (IQ3A).

Synthesis of 2-(2-bromoacetamido)benzoic acid (4). A solution of anthranilic acid (10 g, 72.9 mmol) in a mixture of 1,4-dioxane and DMF (60 mL, 1:1, v/v) was cooled to 0 °C. Bromoacetyl bromide (8 mL, 1.25 equivalents, 91.1 mmol) was added drop wise, keeping the reaction

temperature between 0 and 5 °C. The reaction mixture was then slowly warmed to room temperature and stirred overnight. Ice - water (600 mL) was added, and the precipitate was filtered to give the desired product (18.6 g, 99% yield, mp = 163-165 °C); ^{1}H NMR (DMSO), δ_H (ppm): 11.19 (s, NH), 8.05 (d, *J*= 8.1 Hz, 1H), 7.61 (d, *J*= 8.1 Hz, 1H), 7.23 (dd, *J*= 8.1, 1.5 Hz, 1H), 6.84 (dd, *J*= 8.1, 1.5 Hz, 1H), 3.86 (s, 2H).

Synthesis of 2-(2-((4-(ethoxycarbonyl)phenyl)amino)acetamido)benzoic acid (5). A solution of compound **4** (3g, 11.6 mmol) and 40.7 mmol (3.5 eq.) of 4-ethylaminobenzoate in 50 mL of DMF was placed in a microwave oven and irradiated at 300 W, 140 ºC, for 4h. After cooling, the reaction mixture was poured into 300 mL of iced water and pH adjusted to 8-10 with NaOH 10% (p/v). Then the aqueous reaction mixture was washed with 3 x 100 mL of diethyl ether. The aqueous layer was then acidified to pH=1 by addition of HBr 10% and the product is filtered and dried as a beige precipitate (2.45g, 62 %, mp = 190-193 °C). ^{1}H NMR (DMSO), δ_H (ppm): 11.99 (s, NH), 8.79 (d, *J*= 7.8 Hz, 1H), 8.02 (d, *J*= 7.8 Hz, 1H), 7.82 (d, *J*= 8.6 Hz, 2H), 7.70 (t, *J*= 7.8 Hz, 1H), 7.40 (t, *J*= 4.9 Hz, NH) 7.24 (t, *J*= 7.8, Hz, 1H), 6.75 (d, *J*= 8.6 Hz, 2H), 4.30 (q, *J*= 7.1, 2H), 4.06 (d, *J*= 4.9, 2H), 1.35 (t, *J*= 7.1 Hz, 3H).

Synthesis of 11-oxo-10,11-dihydro-5H-indolo[3,2-*b*]quinoline- 7 carboxylic acid (6). Compound **5** (2g, 7.14 mmol) was heated with 60 g of poliphosphoric acid (PPA) at 130 ºC for 2 hours. After this time the reaction mixture was added to 800 mL of distilled water and basified to pH= 4 with a saturated solution of KOH. Isolation of the crude product was done by extraction with 4x 200 mL of ethyl acetate. The organic layers were dried with anhydrous sodium sulphate and the solvent evaporated under reduced pressure to give a yellow solid (0.337g, 21 %, mp = 294-297 °C). ^{1}H NMR (DMSO), δ_H (ppm): 12.71 (s, NH), 12.12 (s, NH), 8.99 (s, 1H), 8.34 (d, *J*= 7.5 Hz, 1H), 8.03 (d, *J*= 8.7 Hz, 1H), 7.72 (m, 2H), 7.57 (d, *J*= 8.7 Hz, 1H), 7.31 (d, *J*= 7.5 Hz, 1H).

General procedure A. To compound **6** (0.25 mmol or 0.35 mmol) in dried acetone (25 mL), was added 6 equivalents of the corresponding chloroalkylamine and K_2CO_3 (15 eq.). The mixture was heated under reflux for 22 to 46 hours. Reaction was controlled by TLC and at the end, solvent was evaporated and the remaining solid suspended in water (30 mL) and extracted with DCM (3 x 30 mL) to obtain the crude mixture. Pure products were obtained as light yellow oils by preparative TLC. To obtain the compounds as chloride salts, the oils were dissolved in 2 mL of DCM and HCl in ethyl ether was added until precipitation. The solvents were evaporated and the light yellow crystals were dried.

2-(Diethylamino)ethyl 11-(2-(diethylamino)ethoxy)-10-(2-(diethylamino)ethyl)-10*H* indolo[3,2-*b*]quinoline-7-carboxylate (1a) and 2-(diethylamino)ethyl 5,10-bis(2-(diethylamino)ethyl)-11-oxo-10,11-dihydro-5*H*-indolo[3,2-*b*] quinoline-7-carboxylate (2a). Compounds **1a** and **2a** were obtained from reaction of **6** (70 mg; 0.25 mmol) with 2-chloro-(N,N-diethyl)ethylamine hydrochloride according to the General Procedure A, in 13 % yield

(18.5 mg). mp (salt) =134-138 ºC. ^{1}H NMR ($CDCl_3$), δ_H (ppm): 9.22 (d, *J* = 1.5 Hz, 1H), 8.37 (d, *J* = 8.3 Hz, 1H), 8.33 (dd, *J* = 7.8, 1.5 Hz, 1H), 8.31 (d, *J* = 6.3 Hz, 1H), 7.68 (dd, *J* = 8.3, 6.3 Hz, 1H), 7.57 (dd, *J* = 8.3, 6.8 Hz, 1H), 7.49 (d, *J* = 7.8 Hz, 1H), 4.70 (t, *J* = 7.2, Hz, 2H), 4.47 (t, *J* = 6.6, Hz, 2H), 4.29 (t, *J* = 6.2 Hz, 2H), 3.05 (t, *J* = 6.2 Hz, 2H), 2.94 (t, *J* = 6.6, Hz, 2H), 2.80 (t, *J* = 7.2, Hz, 2H), 2.67 (m, 8H), 2.55 (q, *J* = 7.1, Hz, 4H), 1.10 (m, 12 H), 0.91 (t, *J* = 7.1, Hz, 6H). ^{13}C NMR ($CDCl_3$), δ_C (ppm): 166.9, 148.2, 147.5, 146.4, 145.3, 131.3, 129.6, 127.3, 125.4, 125.2, 124.5, 122.5, 122.3, 121.9, 121.5, 109.1, 74.9, 63.0, 53.1, 52.1, 51.2, 47.9, 47.8, 47.7, 44.0, 29.8, 21.5, 12.1, 12.0, 11.9. Compound **2a** was obtained in 14 % yield (20.4 mg); mp (salt) = 175-179ºC. ^{1}H NMR ($CDCl_3$), δ_H (ppm): 8.98 (s, 1H), 8.62 (d, *J* = 8.1 Hz, 1H), 8.16 (d, *J* = 9.0 Hz, 1H), 7.69 (m, 2H), 7.57 (d, *J* = 9.0 Hz, 1H), 7.33 (m, 1H), 4.97 (t, *J* = 7.4 Hz, 2H), 4.84 (t, *J* = 7.5 Hz, 2H), 4.44 (t, *J* = 6.5 Hz, 2H), 3.01 (t, *J* = 7.5 Hz, 2H), 2.92 (t, *J* = 7.4 Hz, 2H), 2.89 (t, *J* = 6.5 Hz, 2H), 2.72 (m, 4H), 2.65 (m, 8H), 1.07 (m, 12H), 1.01 (m, 6H). ^{13}C NMR ($CDCl_3$), δ_C (ppm): 169.1, 166.8, 141.7, 139.7, 131.7, 131.3, 128.1, 126.8, 125.8, 125.1, 123.2, 121.6, 121.4, 114.9, 114.4, 110.4, 63.2, 53.1, 51.2, 50.8, 47.8, 47.6, 47.5, 47.3, 43.4, 12.0, 11.9, 11.8.

2-(pyrrolidin-1-yl)ethyl 11-(2-pyrrolidin-1-yl)ethoxy)-10-(2-pyrrolidin-1-yl)ethyl)-10*H*-indolo[3,2-*b*]quinoline-7-carboxylate (1b) and 2-(pyrrolidin-1-yl)ethyl 11-oxo-5,10-bis(2-pyrrolidin-1-yl)ethyl)-10-11-dihydro- 5*H*- indolo[3,2-*b*] quinoline-7-carboxylate (2b). Compounds **1b** and **2b** were obtained from reaction of **6** (71 mg; 0.25 mmol) with 1-(2-chloroethyl)pyrrolidine hydrochloride according to General Procedure A, in 4% yield (**1b**, 8.8 mg); mp (salt) = 159-164 °C. ^{1}H NMR ($CDCl_3$), δ_H (ppm): 9.23 (s, 1H), 8.32-8.29 (m, 3H), 7.69 (dd, *J* = 7.6, 7.0 Hz, 1H), 7.57 (dd, *J* = 7.6, 7.0 Hz, 1H), 7.52 (d, *J* = 8.6 Hz, 1H), 4.80 (t, *J* = 7.5 Hz, 2H), 4.53 (t, *J* = 6.2 Hz, 2H), 4.38 (t, *J* = 5.8 Hz, 2H), 3.10 (t, *J* = 5.8 Hz, 2H), 2.95 (t, *J* = 6.2 Hz, 2H), 2.89 (t, *J* = 7.5 Hz, 2H), 2.67 (m, 8H), 2.61 (m, 4H), 1.83 (m, 8H), 1.78 (m, 4H). ^{13}C NMR ($CDCl_3$), δ_C (ppm): 166.9, 148.2, 147.3, 146.5, 145.2, 131.5, 129.6, 127.3, 125.31, 125.29, 124.5, 122.5, 122.3, 122.1, 121.4, 108.9, 75.1, 64.1, 55.9, 54.9, 54.8, 54.7, 54.6, 44.4, 23.7, 23.7, 23.7. Compound **2b** was obtained in 6 % yield (8.3 mg); mp (salt) = 183-186 °C. ^{1}H NMR ($CDCl_3$), δ_H (ppm): 9.04 (s, 1H), 8.65 (d, *J* = 7.9 Hz, 1H), 8.20 (d, *J* = 9.0 Hz, 1H), 7.74 (m, 2H), 7.63 (d, *J* = 9.0 Hz, 1H), 7.37 (ddd, *J* = 7.9, 3.4, 3.4 Hz, 1H), 5.09 (t, *J* = 7.7 Hz, 2H), 4.93 (t, *J* = 8.1 Hz, 2H), 4.52 (t, *J* = 6.2 Hz, 2H), 3.15 (t, *J* = 8.1 Hz, 2H), 3.02 (t, *J* = 7.7 Hz, 2H), 2.92 (t, *J* = 6.2 Hz, 2H), 2.82 (m, 4H), 2.74 (m, 4H), 2.65 (m, 4H), 1.92 (m, 4H), 1.82 (m, 8H). ^{13}C NMR ($CDCl_3$), δ_C (ppm): 169.3, 166.9, 141.7, 139.8, 132.1, 131.6, 128.5, 127.0, 126.0, 125.4, 123.3, 121.9, 121.7, 115.1, 114.4, 110.4, 64.2, 56.1, 54.9, 54.8, 54.7, 54.5, 53.2, 47.6, 44.2, 23.9, 23.7, 23.7.

2-morpholinoethyl 11-(2-morpholinoethoxy)-10-(2-morpholinoethyl)-10*H*- indolo[3,2-*b*] quinoline-7-carboxylate (1c) and 2-morpholinoethyl 5,10-bis(2-morpholinoethyl)-11-oxo-10,11-dihydro-5*H*-indolo[3,2-b]quinoline-7-carboxylate (2c). Compounds **1c** and **2c** were obtained by reaction of **6** (100 mg; 0.35 mmol) with 4-(2-chloroethyl)morpholine hydrochloride, according to General Procedure A, in 5 % yield (**1c**, 12.1 mg); mp (salt) = 141-145 °C. ^{1}H NMR (400 MHz, $CDCl_3$), δ_H (ppm): 9.21 (s, 1H), 8.33 (m, 3H), 7.70 (dd, *J* = 7.6, 7.0 Hz, 1H), 7.58 (dd, *J* = 7.6, 7.0 Hz, 1H), 7.48 (d, *J* = 7.8 Hz, 1H), 4.78 (t, *J* = 6.7 Hz, 2H), 4.52 (t, *J* = 6.1 Hz, 2H), 4.36 (t, *J* = 5.2 Hz, 2H), 3.74 (m, 8H), 3.54 (m, 4H), 2.92 (t, *J* = 5.2 Hz, 2H), 2.84 (t, *J* = 6.1 Hz, 2H), 2.75 (t, *J* = 7.8 Hz, 2H), 2.60 (m, 8H), 2.46 (m, 4H). ^{13}C NMR ($CDCl_3$), δ_C (ppm): 166.8, 148.3, 147.5, 146.5, 145.2, 131.4, 129.7, 127.5, 125.4, 124.5, 122.4, 122.2, 121.3, 109.0, 73.2,

67.10, 67.05, 67.00, 62.3, 58.7, 57.5, 54.3, 54.1, 42.9. Compound **2c** was obtained in 11 % yield (25.3 mg); m.p. (salt) 168-170 °C. ^{1}H NMR ($CDCl_3$), δ_H (ppm): 9.00 (s, 1H), 8.63 (d, *J* = 8.1 Hz, 1H), 8.15 (d, *J* = 9.1 Hz, 1H), 7.71 (m, 2H), 7.53 (d, *J* = 9.1 Hz, 1H), 7.36 (dd, *J* = 8.1, 7.0 Hz, 1H), 5.01 (br, 2H), 4.89 (br, 2H), 4.51 (t, *J* = 6.1, Hz, 2H), 3.79 (m, 4H), 3.73 (m, 4H), 3.64 (m, 4H), 3.01 (t, *J* = 7.0, Hz, 2H), 2.82 (m, 4H), 2.69 (m, 4H), 2.58 (m, 8H). ^{13}C NMR ($CDCl_3$), δ_C (ppm): 169. 3, 166.7, 141.7, 139.8, 132.1, 131.4, 128.0, 126.9, 126.1, 125.3, 123.2, 121.8, 121.7, 115.1, 114.3, 110.3, 67.1, 67.0, 62.4, 58.5, 57.4, 55.9, 54.3, 54.1, 54.0, 46.4, 42.6.

2-(piperidin-1-yl)ethyl 11-(2piperidin-1-yl)ethoxy)-10-(2-piperidin-1-yl)ethyl)-10*H*-indolo [3,2-*b*]quinoline-7-carboxylate (1d) and 3-(1-piperidin-1-yl)ethyl 11-oxo-5,10-bis(2-piperidin-1-yl)ethyl)-10,11-dihydro-5*H*-indolo[3,2-*b*]quinoline-7-carboxylate (2d). Compounds **1d** and **2d** were obtained from reaction of **6** (69.7 mg; 0.25 mmol) with 1-(3-chloropropyl)piperidine hydrochloride, according to general procedure A, in 11 % yield (**1d**, 17.7 mg); mp (salt) = 124-128 °C. ^{1}H NMR ($CDCl_3$), δ_H (ppm): 9.21 (d, *J* = 1.7 Hz, 1H), 8.32 - 8.30 (m, 3H), 7.68 (dd, *J* = 8.7, 7.5 Hz, 1H), 7.56 (m, 2H), 4.63 (t, *J* = 6.6 Hz, 2H), 4.42 (t, *J* = 6.5 Hz, 2H), 4.28 (t, *J* = 6.6 Hz, 2H), 2.67 (t, *J* = 6.6 Hz, 2H), 2.55 (m, 2H), 2.47 (m, 8H), 2.23 (m, 8H), 2.05 (m, 4H), 1.63 (m, 6H), 1.54-1.41 (m, 12H). ^{13}C NMR ($CDCl_3$), δ_C (ppm): 167.1, 148.3, 147.5, 146.4, 145.3, 131.2, 129.6, 127.2, 125.3, 125.1, 124.3, 122.6, 122.0, 122.0, 121.5, 109.2, 74.6, 63.7, 56.1, 56.0, 55.4, 54.8, 54.7, 54.6, 43.1, 27.7, 27.1, 26.5, 26.2, 26.0, 25.9, 24.6, 24.5, 24.4. Compound **2d** was obtained in 22 % yield (36.6 mg); mp (salt) = 168-170 ºC. ^{1}H NMR ($CDCl_3$), δ_H (ppm): 8.85 (s, 1H), 8.64 (dd, *J* = 7.8, 1.5 Hz, 1H), 8.14 (d, *J* = 9.0 Hz, 1H), 7.92 (d, *J* = 8.8 Hz, 1H), 7.71 (dd, *J* = 8.8, 7.8 Hz, 1H), 7.67 (d, *J* = 9.0 Hz, 1H), 7.35 (dd, *J* = 9.0, 7.8 Hz, 1H), 4.96 (t, *J* = 6.8 Hz, 2H), 4.85 (t, *J* = 7.3 Hz, 2H), 4.42 (t, *J* = 7.0 Hz, 2H), 2.56 - 2.51 (m, 4H), 2.44 (m, 8H), 2.37 (m, 6H), 2.26 (quint, *J* = 7.3 Hz, 2H), 2.15 (quint, *J* = 6.8 Hz, 2H), 2.04 (quint, *J* = 7.0 Hz, 2H), 1.61 (m, 12H), 1.45 (m, 6H). ^{13}C NMR ($CDCl_3$), δ_C (ppm): 169.2, 167.1, 141.8, 140.0, 131.7, 131.5, 127.8, 126.8, 125.9, 125.3, 123.4, 121.52, 121.47, 114.9, 114.8, 110.7, 63.8, 56.1, 55.8, 55.6, 54.82, 54.79, 54.5, 45.9, 43.2, 28.1, 26.7, 26.2, 26.03, 25.96, 24.6, 24.5.
